# Visual-related conflicts in close relationships

**DOI:** 10.1177/14703572231213936

**Published:** 2024-03-08

**Authors:** Federico Lucchesi, Katharina Lobinger

**Affiliations:** Institute of Digital Technologies for Communication (ITDxC), USI Università della Svizzera italiana, Switzerland

**Keywords:** close relationships, interpersonal conflict, visual communication, visual conflicts, visual practices

## Abstract

This study explores visual-related conflicts, that is, interpersonal conflicts arising from the problematic use of visual communication and visual practices in close relationships. A total of 90 semi-structured pair and individual in-depth interviews with romantic partners and friends were conducted by applying a repertoire-oriented approach. The article explores how the polysemic nature of visuals and different visual practices (e.g. sharing, archiving and deleting visuals), especially related to mundane everyday visual content, contribute to conflictual situations among partners and friends. Specifically, the results highlight that visual-related conflicts occur around miscommunication through interpersonal communication, non-negotiation around visual sharing, not including partners in online relational presentations, online monitoring activities on social network sites and intrusive requests to delete visuals. This study extends the understanding of potential risks to close relationships from problematic uses of visual communication.

## Introduction

All interpersonal relationships, especially close relationships such as romantic relationships and friendships, experience moments of interpersonal conflict (Brehm et al., 2001; Hocker and Wilmot, 2018). Conflict sources tend to vary as conflicts can arise due to any form of disagreement regarding goals, aspirations, values and everyday life issues (Azim, 2017; Putnam and Poole, 1987). Communication research has found that conflicts arise during face-to-face encounters, mediated communication, or are due to the use of technology or specific platforms, for example, social network sites (SNSs). Although conflicts are often addressed with a negative normative connotation, they do not necessarily harm the relationship. Rather, recognizing the source of a conflict, communicating properly and achieving positive conflict resolution are fruitful for maintaining close relationships (Wilmot, 1995).

With the advent of networked visual technologies and the increased diffusion of social media (Leaver et al., 2020), visual communication and visual practices have become networked and thus embedded in globally connected information flows (Lister, 2007). Visuals are also central to maintaining close relationships (Keightley and Pickering, 2014) and can stimulate immediate emotional responses (Harper, 2002). These aspects have also been found to elicit positive emotions and shared memories (Prieto-Blanco, 2016), which can help foster closeness (Lobinger et al., 2021). In this article, we argue that visual communication and visual practices can also contribute to interpersonal conflict, which we define as conflicts that arise around visuals, that is, ‘visual-related conflicts’. While studies have found connections between online visual sharing and adverse relational outcomes (e.g. Muscanell et al., 2013) or between visual sexting and harmful consequences (Chalfen, 2009; Thorhauge et al., 2020; Thurlow, 2017), a broader focus on visual communication and visual practices as sources of conflict is currently missing. Identifying and understanding visual-related conflicts is particularly relevant in terms of deepening our understanding of the role of visual communication in close relationships. Understanding this role can provide insight into whether and how visuals, by their very nature, engender conflict situations that would otherwise not arise. Accordingly, the article addresses the following research question (RQ): Which visual-related conflicts occur in close relationships, and how do they occur? Through this research focus, we aim to expand the understanding of the problematic uses of visual communication when it comes to analysing close relationships.

The present study is part of a larger research project examining the functions and social norms related to visual communication in close relationships in Switzerland. Through 90 semi-structured interviews with romantic partners and close friends, we asked our respondents to reflect on the sources and development of visual-related conflicts while exploring their communication repertoire, visual practices and use of visual technologies. The current article starts with a theoretical introduction, defining interpersonal conflicts and their role in maintaining close relationships. We then outline how previous communication research has investigated mediated communication, the use of technology as sources of conflict and the relationship between visuals and conflicts. Subsequently, we describe our methodology. In the results section, we present the different sources of visual-related conflicts emerging from our analysis. Finally, we discuss our conclusions and suggest directions for further research.

### Interpersonal conflict in close relationships

Close relationships are understood as interpersonal relationships characterized by an intimate bond and intense emotions (Blumstein and Kollock, 1988). Regardless of the people involved (e.g. friends, partners, family), the key distinguishing trait of such relationships is a ‘strong, frequent, and diverse interdependence that lasts over a considerable time’ (Kelley et al., 1983: 38). Interdependence is formed through the constant interactions that occur between the members of the close relationship over time (VanderDrift and Agnew, 2019) and is based on shared relational experiences (Reis et al., 2000). Concretely, interdependence is manifested through mutual behaviours or attitudes and the establishment of norms or expectations cultivated within the relationship (Regan, 2011), which contribute to the bond.

Interpersonal conflict is defined as ‘an expressed struggle between at least two independent parties who perceive incompatible goals, scarce resources, and interference from the other party in achieving their goals’ (Hocker and Wilmot, 2018: 3). The interdependence that characterizes close relationships makes such conflicts more likely than in other interpersonal relationships (Braiker and Kelley, 1979) because every decision made by one individual also impacts the other person (Bateson, 1972). Members of close relationships must balance the need for independence (individual goals) while cooperating to pursue relational goals (Leo et al., 2019) – a dialectic that facilitates the emergence of conflicts (Putnam and Poole, 1987).

Conflicts may arise because people manifest less concern, love, care, or empathy (Gordon and Chen, 2014). Additional reasons can be a reduction in mutual interest (Sinclair and Fehr, 2005), insecurity or jealousy (DiBello et al., 2015), or infidelity (Hall and Fincham, 2006). Conflicts commonly follow a situation perceived as a form of betrayal (Leary et al., 1998), for example, extradyadic sexual relations or sharing information meant to be private (Feeney, 2004). In these cases, relational rules or expectations are violated (Caughlin et al., 2009). However, conflict can also occur when two people disagree on something and fail to take steps toward the other’s position (Huang, 2010), as can happen, especially around topics like communication, finances, parenting and sex (Meyer and Sledge, 2022). We argue that conflicts provide a research opportunity since they shed light on broken norms or rules that can otherwise go unnoticed.

Although we often refer to conflict in negative terms, it is an integral part of any healthy relationship (Eidelson and Epstein, 1982) and is neither categorically beneficial nor detrimental. Instead, how it is managed determines whether the consequences will be positive or negative (Gross and Guerrero, 2000; Yildiz, 2023). Indeed, relationship maintenance largely depends on proper conflict management (Canary and Stafford, 1992). People should adopt a dyadic perspective when communicating during a conflict to promote a better mutual understanding (Meier et al., 2021). Through conflict, two people can discuss their needs and desires within the relationship (Burgess and Burgess, 1996), handle situations of disagreement and prevent them from recurring in the future (Wilmot, 1995).

A positive conflict outcome can facilitate communication between those involved (Benjamin, 1990), enhance relationship quality (Mandal and Lip, 2022) and develop greater closeness among the parties (Siegert and Stamp, 1994). Conversely, a negative conflict outcome can generate depression and anxiety (Koerner and Fitzpatrick, 1997), anger and grief (Sereno et al., 1987), relational dissatisfaction (Todorov et al., 2021) and even violence (Infante et al., 1990). As discussed in the next section, communication scholars have shown that the use of technology or certain communication channels can contribute to interpersonal conflicts.

### Mediated communication and interpersonal conflicts

Interpersonal communication in close relationships is increasingly mediated (Hepp and Krotz, 2014), and information and communication technologies have facilitated interactions in maintaining personal connections (Baym, 2010). Conflicts can occur and be managed during face-to-face interaction and in mediated settings (Perry and Werner-Wilson, 2011). As such, according to the communication interdependence perspective (Caughlin et al., 2016), face-to-face and mediated communication are not adopted as separate entities but are inevitably intertwined in close relationships. For instance, a conflictual issue can be discussed repeatedly over time and each time it can involve various communication channels.

Communication research has investigated mediated communication, the use of technology and specific platforms such as SNSs as possible sources of conflict. For instance, a study by Duran et al. (2011) found that most conflicts related to smartphones are linked to the relational dialectic of autonomy versus connection (Baxter and Montgomery, 1996). Since ubiquitous technology enables forms of ‘perpetual contact’ (Katz and Aakhus, 2002), an excessive amount of daily interaction or poor responsiveness when contacting the other can generate conflict (Baron, 2008). Likewise, a widespread source of conflict that has a negative impact on relationship satisfaction (Beukeboom and Pollmann, 2021) is *phubbing* (Roberts and David, 2016), which is where a device such as a smartphone is used while in the company of another person, who consequently feels ignored and deprioritized (MacDaniel and Coyne, 2016).

Furthermore, research has highlighted that the use of SNSs can make close relationships vulnerable (Abbasi, 2019). One study even coined the term *Facebook-related conflict* to define aspects of SNS use that can generate interpersonal conflict (Clayton et al., 2013). High SNS use has been linked to undesirable consequences such as emotional and sexual infidelity, relational dissatisfaction, lower commitment and breakups (Bouffard et al., 2022; Drouin et al., 2014; Utz and Beukeboom, 2011). However, one of the primary sources of conflict due to SNS use is the management of relational presentations. SNSs are often used by individuals seeking visibility (Marwick and boyd, 2011) and to convey a positive self-image (Zhao et al., 2008), usually through the concealment of unattractive content that could be perceived negatively by the audience (Walther et al., 2008). For this purpose, people in close relationships can disagree about relational presentation strategies when publishing on SNSs, such as (not) sharing status updates or couple pictures (e.g. Papp et al., 2012). A second major source of conflict around SNSs is the peer-to-peer surveillance of the other’s online activities, that is known as *partner monitoring* (Darvell et al., 2011) when referring to a romantic partner and as *lateral surveillance* (Andrejevic, 2004) or *social surveillance* (Marwick, 2012) when also including other kinds of close relationships such as relatives or friends.

Several studies have reported that partners and friends use SNSs to gather information about each other and that the information uncovered, or the surveillance activity itself, can arouse jealousy and conflict (e.g. Arikewuyo et al., 2022; Marshall et al., 2013). Since conflict can occur in various forms, adopting a communicative interdependence perspective (Caughlin et al., 2016) can help highlight how it can emerge and be addressed through a combination of communication channels used on distinct occasions by the people involved.

### Toward the concept of ‘visual-related conflicts’

With the digitization of photography, mediated communication has increasingly been based on visual communication (Rubinstein and Sluis, 2008). In this article, visual communication is understood as ‘the circulation of non-linguistic pictorial elements that feature in cultural artifacts distributed via media technologies’ (Aiello and Parry, 2020: 4). With the spread and convergence of visual and networked technologies, such as networked camera-equipped smartphones available at one’s fingertips at any time, producing and sharing visual content of everyday life have become ordinary activities (Frosh, 2003; Hand, 2012).

In his work on media practices, Couldry (2004: 119) defined practices as what ‘people are doing in relation to media across a whole range of situations and contexts’. Similarly, in this article, we are interested in understanding what people in close relationships are *doing* with analog and digital non-linguistic elements (e.g. pictures, videos and GIFs), which we refer to under the broad term of *visuals*. Thus, we speak of *visual practices*. The emphasis on visual practices means that we consider both the content and materiality of visuals (Siles and Boczkowski, 2012), within the practices and in the context of the multi-modal visual technologies through which visuals are created and utilized. In other words, we understand visuals ‘beyond what a photograph’s surface visually displays’ (Edwards, 2012: 224). Examples of potential *visual practices* include sharing, archiving, editing, displaying, or deleting visuals. It is important to consider that visual practices can only be understood within the specific social setting of close relationships (Aiello and Parry, 2020).

Visual communication and visual practices can help foster intimacy (Lobinger et al., 2021; Miguel, 2016) and build closeness while developing interdependency (Hatfield, 1984). Furthermore, visual communication has been attributed important social functions for relationship maintenance (Van House et al., 2005), and the holistic character of visuals (Müller, 2007) facilitates the conveyance and elicitation of emotional disclosure (Berger, 1982). Usually, disclosure, intimacy and conflict are intertwined (Sandhya, 2009) and sometimes an increase in perceived intimacy can go together with an increase in conflict (Seiffge-Krenke, 1997). In other words, the centrality of visual communication and visual practices within everyday life and close relationships and the role of visuals in fostering intimacy suggest that visuals may also become a source of interpersonal conflict.

Several studies on close relationships have marginally included visuals in their analysis as potential sources of adverse relational outcomes. Existing findings suggest that uploading pictures on SNSs can be perceived as a privacy invasion (Teutsch et al., 2018) and that updating profile pictures on SNSs can negatively impact relational satisfaction (Muscanell et al., 2013) or engender jealousy (Muise et al., 2014).

However, to our knowledge, only one study has explicitly examined the relationship between visuals and conflict. Such et al. (2017: 3821) investigated conflicts that occur around photo-sharing practices on SNSs when ‘the privacy preferences of the uploader and co-owners of an item do not align’. They found that conflicts about sharing photographs arise especially in close relationships because partners and friends are perceived as more trustworthy than weak ties, and thus send and receive more ‘sensitive’ pictures. On one hand, because of trustworthiness, close ties do not feel the need to establish clear rules regarding the use of visuals. However, a lack of rules creates the conditions for potential conflict (Venema and Lobinger, 2017). On the other hand, rules can be negotiated as a preventative strategy to avoid conflict, albeit without always finding a compromise between different positions (Such et al., 2017).

In this article, we investigate how the use of *visual communication* and *visual practices* can engender interpersonal conflict. To do so, we adopt a communicative interdependence perspective and argue that studying the entire communication repertoire of a close relationship makes it possible to acquire contextual information and reach a more complex understanding of how visual-related conflicts arise.

## Method

The present study is part of a larger research project exploring (a) the role and functions of visual communication among partners and friends in Switzerland, and (b) which rules and norms of visual communication are established within such close relationships. Between September 2019 and July 2021, we conducted 90 semi-structured in-depth interviews with 21 dyads of romantic partners and nine close friendship dyads in Switzerland (60 adults, 18–91 years, *M* = 36.31).^1^ The interviews were conducted in (Swiss) German, French, Italian and English. In selecting the participants, we aimed for diversity regarding the region of origin, age, education levels, profession, length of the relationship, status and housing situation. We included couples with and without children, and same-sex and heterosexual relationships.

We first conducted a semi-structured in-depth pair interview, interviewing two romantic partners or close friends together (Longhurst, 2009). After about two weeks, we conducted individual interviews with the two members of the dyad. Therefore, each respondent was interviewed twice, allowing both individual and couple perspectives to be captured. The average interview length was around 90 minutes.

Regarding the aspect of conflicts, we aimed to understand how visual conflicts arose and how they were addressed and resolved by the respondents. To prompt discussion about potential disagreements, we investigated the norms, rules and roles that the dyads established for visual practices, such as taking, sharing, archiving, or posting visuals online. In addition, we explored the relational communication repertoires from a communicative interdependence perspective by asking the respondents to outline their ‘communication universe’, that is, to create a network drawing (Hepp et al., 2016) that included all the communication channels they used and describe the role and function of the visuals within it. Adopting this repertoire-oriented approach (Linke, 2011) allowed us to understand the situations in which conflicts arose and how different communication channels were combined to address them. We were interested not only in major conflicts in crisis situations but also minor misunderstandings and disagreements, that is, everyday conflict situations that require confrontation and negotiation to be resolved. First, the collected data were manually transcribed, then we performed a thematic analysis (Kuckartz, 2014) using the NVivo software to gather information about visual-related conflicts. For this purpose, we manually coded the interview transcripts through a combination of inductive and deductive categorizations. At this stage, two researchers conducted an ongoing discussion to create and revise a codebook collaboratively. Finally, we created case summaries (each case including one pair interview and two individual interviews) as helping tools for cross-case coding and analysis to reveal similarities and discrepancies regarding conflict-related data.

## Results

Our research question aimed to explore which visual-related conflicts occurred in close relationships and how they emerged. The results revealed that various interpersonal conflicts arose due to visual practices and visual communication, and that they occurred within the context of a heavily mediated communication repertoire that is typical of close relationships. In the following paragraphs, we outline the visual-related conflicts experienced by the partners and friends, and provide insight into how the specific nature of visuals can yield interpersonal conflict.

### Miscommunication through visual communication

Except for very few dyads, messaging platforms such as WhatsApp were central components of the communication repertoires. Partners and friends reported increased use of such channels for visual interpersonal communication. While exchanging visuals on messaging platforms, our respondents reported several cases of miscommunication that created minor visual-related conflicts related to the visual modality. With respect to interpersonal communication, *miscommunication* is commonly defined as the inability of partners to establish shared interactive meaning (Mortensen, 1997). The fact that images and their exchange are a source of misunderstanding can be explained by the polysemic nature of images. While the denotative motif of a visual may be clear to both conversational partners, its connotative meaning depends on the (often different) knowledge and interpretation of the conversational partners. However, most of our interviewees underestimated the chances of miscommunication when communicating visually and considered visuals to be more straightforward and less open to interpretation than written text. ‘*With images, it is harder not to understand the message*’, laughed Tommaso (male, 33 years) ‘*whereas when I write, then yes, I write “Roma per Toma”*,^2^ *and we never understand each other*.’ Carolina (female, 19 years) believed that ‘*the photograph is either like this or like that; you cannot interpret it . . . because he sees just what I see*.’^3^

The respondents underestimated the extent to which their partner might attribute a different meaning to a photograph and thus to an entire conversation. This lack of awareness of the polysemic nature of an image created fertile ground for conflict. For example, Dennis (male, 35 years) described an incident when his wife, Valentina (female, 30 years), decided to go to the gym, taking her feverish young daughter with her and leaving her on a mat to sleep. She then sent the photo of their daughter to Dennis, who reacted angrily. ‘*If you send me a picture of my daughter sleeping on the mat, it does not make me tender; it pisses me off because she should sleep in her bed.*’ What to Valentina^4^ was a sweet portrait – the baby sleeping snuggled up – to Dennis was irresponsible. ‘*If she is not well, couldn’t you have stayed home*?’, Dennis replied. In such a situation, seeing the scene through the photograph made Dennis fully aware of what was happening, arousing negative emotions and triggering conflict.

Gaining awareness of the polysemic nature of visuals can help make more conscious use of images but does not always prevent miscommunication and subsequent conflict. Alberto (male, 25 years) explained that he wanted to tease his partner erotically and sent a provocative meme to her (Costanza, female, 25 years). ‘*You can find an image that can have a double meaning, and you send it on purpose*’, Alberto said. Although he considered such playful use of images appropriate for flirtation, Alberto underestimated the highly contextual meaning, and his girlfriend perceived his message as obscene. ‘*You do not know with which tone the other person is communicating*’, Costanza explained. Overall, the above-mentioned incidents suggest that it is crucial to consider contextual aspects in order to reach a shared understanding of a visual.

### (Non)negotiations about visual sharing and (moral) ownership of visuals

Another source of conflict concerned (non)negotiations about online visual sharing. Our respondents often took the perceived (moral) ownership of visuals for granted until disagreements emerged from a specific episode. They were aware that visual technologies had expanded the possibilities for circulating and editing visuals and often expressed a desire to keep personal information, such as pictures, under control. A lack of control is particularly problematic and prompts discussions about moral ownership of visuals, that is, who has the ‘moral right’ to use and share a visual and who does not.

To avoid risks associated with online sharing, our respondents agreed that some intimate photographs, such as erotic pictures, must not be shared with third parties for any reason. Sometimes, even the exchange of erotic pictures between the two partners is perceived as inappropriate, as they know that a relationship is an ongoing process that could potentially end. As Zoé (female, 28 years) underlines, ‘*I would not like someone to have naked photos of me, especially if we are not together anymore. I would be afraid* (of what the other could do with her pictures).’ For other photographs labelled ‘sensitive’, for example, those including children or embarrassing pictures of drunk people, sharing with close ties on WhatsApp was perceived as unproblematic, while sharing on SNSs was to be avoided. SNSs are perceived to be ‘riskier’ than messaging apps because the respondents felt that they had less control over the potential online circulation of the pictures they uploaded. When the sharing of ‘sensitive’ pictures occurred, they highlighted that negotiations were fundamental in finding mutual agreement and preventing conflict.

However, when it came to the online sharing of ‘innocuous’ everyday pictures, for example, ‘couple pictures’,^5^ photos of personal items, or those of groups, such negotiations often did not occur. Indeed, all members believed that they knew their partner well enough to be sure about what they would or would not allow them to share. Consequently, establishing norms about sharing photographs was perceived as unnecessary, at least until the first conflict arose.

Conflicts developed on two levels: on one hand, the ‘motif’ of the shared visual was the focus of the dispute, for example, when it was aesthetically displeasing and compromised the ideal of self-presentation of one of the two partners. ‘*I did not like how I looked*’, Yara (female, 25 years) pointed out, commenting on a ‘couple picture’ posted on Instagram by her best friend. ‘*She did not ask me if she could post that picture*’, Dreina (female, 22 years) complained, claiming her right to be involved in sharing decisions. On the other hand, the respondents reported conflicts due to a lack of agreement regarding the (moral) ownership of the shared visual. ‘*I should be aware of the files containing my image, or the image of us as a couple, that are on the net. It is my thing. It is my privacy*’, Alberto complained.

Usually, the two levels are interconnected, as one person’s visual sharing is deemed inappropriate by the second, who then accuses the first of not having the (moral) right to share without consultation. For example, Costanza sent a picture she had found on her boyfriend’s computer to a common friend. She was unaware that Alberto had intended to print, frame and gift that picture to this friend, and Costanza’s decision led to an angry discussion between the partners. Costanza defended her right to share, saying that ‘*I am also in the photograph*’, but Alberto considered the picture his ‘property’ since it was stored on his computer.

We can conclude that (moral) ownership of visuals is a complex and nuanced concept that is not easy to foresee but plays a significant role in the sharing of visuals online. Without proper prior negotiation, the sharing of visuals can become a source of conflict. When this happens, finding agreement despite divergent ownership claims (e.g. eliminating the photograph) is critical to resolving the crisis. These conflicts can also become an impetus for negotiating new social norms about future visual sharing. Importantly, once a new norm is established, it must be respected. According to our respondents, establishing a norm and then failing to abide by it was even more disconcerting as it was perceived as deliberately neglecting the point of view of the other.

### Feeling excluded from the social media life of the other

Not including the partner or friend in pictures posted on SNSs was another source of visual-related conflict because partners and friends expected to be included in online relational presentations. Our respondents emphasized that, when they posted an image online, their primary interest was to convey an aesthetically pleasing image of themselves and that this priority led them to sacrifice the relational presentation. ‘*I had chosen and posted a picture without my best friend, even though I had a version of the picture with him, because I looked ten times better*’, Derrick rationalized (male, 25 years). Some respondents reported more extreme measures, such as editing a picture to eliminate the presence of the other to appear in the exact centre of the shot. However, generally, the excluded person was not conscious of the motivations behind the exclusion and perceived such a choice as a desire to keep the relationship hidden. Chiara (female, 21 years) ‘*became a little jealous*’ when her boyfriend stopped posting couple pictures ‘*because there are girls who are sluts; they try and everything . . . no one knows that we are together if there is no photo proving it publicly*’, she revealed to us. Apart from Chiara’s obvious use of gender stereotypes, she was linking the absence of couple pictures to the possibility of her boyfriend attracting alternative partners on SNSs and was fearful that such absence could undermine their relationship.

Despite the reasons for excluding others from online publications, the behaviour fuels a strong sense of disappointment, and social comparison processes can exacerbate these emotions. Social comparison can feature more generally (‘*I could see all the other couples who had published 200 million photos together*’) or with another relationship. Nola (female, 20 years) told us that her best friend Adelaide (female, 20 years) had stopped publishing pictures with her and had begun posting pictures with another friend. ‘*I did not like to see their photos*’, she explained, because they were evidence that ‘*Adelaide was always with her, whereas before, she was always with me*.’

In other words, our respondents associated relationship quality or ‘existence’ with the presence of couple photographs on SNSs. In such cases, the intervention of external actors further complicated matters and stimulated conflict. Silvestro (male, 36 years) recounted a period of crisis with his partner Giuseppina (female, 35 years). They had almost broken up, and he felt very insecure. ‘*That is when my colleagues told me* . . . “*but how come you do not have pictures* (on SNSs) *with Giuseppina*?”’ By saying this, the colleagues instilled doubt in Silvestro regarding Giuseppina’s supposed desire to keep their relationship hidden, fuelling his insecurities. ‘*They associated this with her not loving me, that she is not interested in me* . . . *that it is not a real relationship*.’ The colleagues’ insinuation bothered Silvestro so much that he went to talk to Giuseppina, and ‘*a heated conflict followed*’.

When such conflicts occurred, a typical solution was to include the partner or friend in SNS publications to compensate for prior exclusion. For instance, Derrick realized that he had hurt his friend. ‘*I made up for it by putting on Facebook this* (another photo in which the friend was also present) *. . . to emphasize that I liked that we had taken it together*.’ However, if after a confrontation one partner keeps excluding the other, some respondents reported adopting similar behaviour, balancing and thus reducing the relational investment in terms of visually representing the relationship.

### Online monitoring and lack of trust

Several visual-related conflicts arose due to unexpected discoveries while observing visual content shared by partners and friends on SNSs. In general, our respondents emphasized that they did not actively monitor their partner’s or friend’s online activity and that recommendations regarding each other’s content depended on platform affordances. ‘*I mean, I do not look for the profile to see if she posted something. Generally, I find something in my Instagram feed, or I have it in front of me in the Stories*’ (Matteo, male, 19 years).

However, the participants normatively associated online monitoring with hostile surveillance, which is why they were keen to emphasize that they did not purposely go in search of content. In fact, several participants pointed out that they did not need to do this because they trusted the other. ‘*I do not look for his content because I trust him . . .*’ (Marianna, female, 31 years).

The concept of trust cited by Marianna plays a crucial role in engendering conflicts. It is not the act of observing published content that is a source of conflict per se; instead, what triggers conflict is the discrepancy between what is observed online and what has previously been reported by the other person. For instance, Vanessa (female, 20 years) was planning to have dinner with two friends but, at the last minute, they informed her that the dinner had been cancelled as they had to prepare for an academic exam. However, she later noticed a picture on Instagram Stories that portrayed her two friends, along with a third person, eating hamburgers. Upon seeking clarification, the friends explained that they had arranged the dinner at the last moment. Nonetheless, the damage was done because Vanessa’s trust in her relationships had been eroded. When trust expectations are not met, a feeling of dissatisfaction or even betrayal is likely to prevail.

According to the romantic partners we interviewed, the chances of generating conflict from online monitoring increase when the visual content shows the other with a potential alternative partner. For instance, Dennis (male, 33 years) grew nervous when his wife went skiing with her brother and his girlfriend. ‘*I saw a picture . . . and there was also a mutual friend. . .*’ Having seen another man in an online picture made Dennis perceive the situation as a double date, eliciting jealousy and provoking conflict. Sometimes, some hidden cues are enough to perceive something as a ‘couple situation’, thereby eliciting conflict. Giorgia (female, 25 years) got into a quarrel with her boyfriend because a mutual friend had uploaded a dinner photograph on Instagram, about which she was unaware:

*It looked like a ‘couple picture’. . . It was that classic shot that you maybe take when you are having dinner with your boyfriend and you want to show the plate . . . There is that element, the see-and-don’t-see, that lets you know who that other person is.*

Finally, also social media reactions (e.g. ‘love’, ‘like’, ‘preferred’ or others) (Scott et al., 2020) can be the subject of online monitoring. These are also known as ‘paralinguistic digital affordances’ (Hayes et al., 2016: 172–173), that is, ‘cues in social media that facilitate communication and interaction without specific language associated with their messages’ and are often used to express appreciation towards online content. Filiberto recounted a moment of serious conflict with Chiara about SNS use: ‘*she comes to me and says “eh, you have liked this one* (i.e. posts by other girls), *that one, etc.*”, *every time.*’ In the meantime, however, Chiara was commenting on male users’ content. Such behaviour created discomfort in Filiberto since she was engaging in *precisely* the behaviour that she had demanded that he discontinue. To avoid further conflict, Filiberto deactivated his Instagram account; however, this created relationship dissatisfaction and imbalance, which is not functional for maintaining a romantic relationship.

### Requests to ‘delete memories’

Finally, visual-related conflicts also emerged due to unreasonable requests to delete pictures. When asked to reflect on the importance of visual elements both individually and for the relationship, our respondents often cited the role of the image in remembering moments or experiences. They explained that they kept photographs to ‘*look at them again*’ because flipping through a photo album or a digital gallery allowed them to relive past emotions and shared experiences. They cared about archiving and images were often printed because of fear that digital photographs might be lost due to technological problems. The respondents stored visuals on multiple fronts, using both hard disks and cloud storage, ‘*because then, maybe, I have problems, and maybe they get deleted, and I lose them*’ (Adelaide).

Given the significance of the mnemonic aspect, a partner’s request to delete personal photographs can be perceived as emotional violence. For instance, Giuseppina and Silvestro sparred over Silvestro’s request that Giuseppina delete all photographs of her former partner. Giuseppina deleted the pictures but perceived Silvestro’s request as inappropriate as he had imposed a decision that should have been hers. ‘*I did so but very unwillingly*’, Giuseppina explained, ‘*they are my photos; I do what I want with them . . . I deleted them, but I regretted it . . . I considered it a very problematic request*.’ Furthermore, it implied unfounded feelings of jealousy as well as trust issues that were detrimental to the relationship. ‘*Afterwards, we talked about it, and I said, ‘Look, I did it now, but honestly, I should not have.*’ This example underscores that, in close relationships, any individual visual practice, such as choosing to safeguard pictures or requesting that they be deleted, is intertwined with personal freedom and relational balance.

## Discussion

Our study investigated specific visual-related conflicts between partners and friends, and how they arise. Against the backdrop of interpersonal communication and different visual practices (e.g. sharing, archiving, deleting visuals), our findings showed how visuals contribute to creating conflictual situations.

The study revealed that, during visual interpersonal communication, misinterpretations can occur due to lack of appropriate reflection, which can be understood in relation to our respondents’ positivist view of photography, that is, the perception of images as a precise, truthful representation of objective reality (Batchen, 1999; Chandler and Livingston, 2016). In other words, individuals ignore that a visual ‘cannot reveal intention, nor can its meaning be fixed, because it is polysemic and formally malleable by design’ (Frosh, 2003: 73). Miscommunication also occurs when individuals are not aligned on the role of visual communication when exchanging visuals. A typical example is when a picture is sent with phatic intention (Niemelä-Nyrhinen and Seppänen, 2020), that is, ‘to share a picture mainly for the sake of visual connectivity’ (Kofoed and Larsen, 2016), while the other focuses on the image motif instead. Moreover, we argue that visual practices are increasingly embedded in mediatized networked practices; thus, they ‘have become a routine rather than . . . isolated incidents of reflexive engagement’ (Hand, 2012: 67). Unlike other modes of communication, such as textual or oral communication, visual modality implies a greater immediacy of communication (Müller, 2007), which could contribute to routinized visual practices without proper reflexive engagement.

Additionally, our findings show that (non)negotiations about online visual sharing play a role in interpersonal conflict. Our respondents were aware that digitalization had changed social norms regarding visual ownership (Heaven, 2013) and expressed concerns about the risk of ‘sensitive’ visuals circulating online without control. Such a danger seems especially valid on platforms such as SNSs, where pictures can easily ‘travel’ through multiple audiences (Baym and boyd, 2012). This echoes previous studies on privacy norms and sexting (Hasinoff and Shepherd, 2014) since norms are established to avoid sharing intimate images such as erotic pictures. Additionally, our respondents appeared to be aware of the dynamic nature of relationships and the potential risks associated with losing control over erotic pictures in the event of a relationship ending (Maddocks, 2018).

Similarly, according to Ranzini et al. (2020), sharing children’s photographs on SNSs is considered highly inappropriate and calls for norms around the practice. Indeed, our respondents did not consider children’s pictures to promote their parental digital self (Blum-Ross and Livingstone, 2017) or build a sense of community with other parents (Bartholomew et al., 2012). Instead, these pictures were considered ‘highly personal’. However, concerning other visual practices, our study confirmed that parents rarely acknowledge children’s agency in advocating for their own privacy (Li et Gui, 2022). For instance, they seldom seek children’s permission when taking pictures of them. Overall, in the case of sensitive pictures, negotiating rules regarding visual practices in advance can avoid interpersonal conflict. Nevertheless, consistent with Venema and Lobinger (2017), in the case of ‘less sensitive’ everyday pictures (e.g. ‘couple pictures’), rules for sharing visuals are often not negotiated because members of close relationships feel that they know each other so well that they do not need norms to decide what to share. In other words, our findings suggest that it is the mundane everyday visual sharing that might create conflict among couples and friends rather than practices considered more ‘sensitive’, such as sexting. The lack of negotiation paves the way for interpersonal conflict when partners and friends disagree about the use of pictures. Indeed, confirming the finding of Such et al. (2017), both the person who takes the photograph and the person portrayed in it can perceive ownership of the (moral) right to choose whether to share it. Thus, when one fails to recognize the other’s (moral) right to share, tension can ensue. This also confirms that agency is not a stable concept in the context of networked visual practices (Velez, 2019). While individuals may exercise agency in deciding to take or be portrayed in a photograph, they might subsequently lack control over the archiving, modification, or circulation of the resulting image. Overall, our examples underline the importance of negotiating rules regarding visual practices within communication repertoires to avoid visual-related conflicts.

It is commonly understood that SNSs present affordances designed for individual use. We contend that this emphasis on the individual aligns with neoliberal ideologies and processes of individualization that predominate in contemporary Western society (Beck and Beck-Gernsheim, 2002; Harvey, 2005). Previous research has highlighted that SNS users value positive visual self-presentation (Zhao et al., 2008), which has a fundamental impact on personal self-esteem (Gonzales and Hancock, 2010). Our study confirms that individuals choose pictures to be published on SNSs based on their physical appearance or personal aesthetics (Fox and Vendemia, 2016). However, it is difficult to consider the individual and the relationship as separate entities, especially in romantic relationships. Consequently, interpersonal conflicts may arise when a visual relational presentation on SNSs is lacking. Indeed, some partners want to show the intimacy of their relationship as part of their self-presentation, and they expect to see ‘couple pictures’ shared by their partners (VanderDrift et al., 2015). Our study not only confirms the importance of visual relational presentation (of some couples) on SNSs in increasing relationship satisfaction in romantic relationships (Cole et al., 2018) but also extends these findings to other close relationships, such as friendships. Overall, our results show that both partners and friends (at least some) feel neglected if they are not represented visually within a relational presentation, which can generate tension and conflict. We argue that, even though the affordances of SNSs might encourage individual representation, the interpersonal dimension of close relationships also needs to be included. However, individuals struggle between opting for individualized uses of SNSs and respecting the expectations of their partner or friend.

Our findings show that monitoring the other on SNSs can lead to unexpected discoveries, which can lead to interpersonal conflict. Since SNSs are increasingly based on visual communication, visuals play a primary role in online monitoring. Previous research has highlighted that jealous individuals adopt online monitoring to actively seek information about their partner (Marshall et al., 2013) and that monitoring a partner on SNSs can have adverse relational outcomes (Arikewuyo et al., 2022). Our study confirms that the use of social media reactions when responding to visuals on SNSs can be interpreted with different meanings by partners and friends (Hayes et al., 2016), contributing to inducing jealousy during online monitoring. Our findings show that online monitoring is an everyday activity among partners and friends. This confirms social surveillance as a practice diffused on SNSs since each user is sharing content that is looked at by others and looking at content published by others (Marwick, 2012). However, online monitoring is not described as a systematic activity, which would be typical of traditional surveillance processes (Lyon, 2007). Furthermore, differently from *social searching* (Lampe et al., 2006), the information discovery does not necessarily happen due to active information searches. For our respondents, looking at the visuals published by the other was not perceived as a surveillance practice but, rather, a way to keep informed about what the other was doing. This is in line with the finding of Marwick (2012), which described this kind of ‘ambient awareness of the other’ as a means of caring about the other and maintaining the relationship through digital platforms. However, despite the idea of ‘careful surveillance’ (Hjorth et al., 2018), such a practice is still characterized negatively, and individuals justify their actions by emphasizing the role played by the platforms’ algorithms in alerting them to visuals posted online by the other.

Additionally, visuals have a personal affective dimension because they elicit personal emotions and feelings (Keightley and Pickering, 2014) that go beyond the dyadic perspective. The spread of visual technologies, such as camera phones, has enabled individuals to take photographs and more easily keep a visual chronology of digital memories of their lives and daily activities (Huang and Hsu, 2006). Our results highlight a highly emotional connection between photography and its mnemonic function (Sontag, 1973), which is critical to an individual and thus a partner’s request to delete pictures can generate interpersonal conflict. In fact, preserving everyday memories is, among other things, functional to a person’s identity construction (Olsson et al., 2008). Therefore, the advent of a close relationship might be problematic when challenging personal memories experienced outside of and prior to a relationship. In the words of Barthes (1980), visual chronologies of the individual are ‘traces’ of a past experience that one may have the desire to preserve.

Taken together, our findings also confirm that close relationships are distinguished by strong interdependence (Braiker and Kelley, 1979) and that every action and choice of one person impacts the other (Bateson, 1972). If a dyad communicates visually or uses visuals by considering each other’s views and expectations and adopting a dyadic lens, then interpersonal conflict can be avoided. If members of close relationships fail to balance individual and relational needs (Putnam and Poole, 1987) in visual practices – being self-focused when using visuals – then interpersonal conflict can arise.

## Conclusion

Conflictual situations are common in close relationships, and recognizing and resolving interpersonal conflict can be critical to relationship maintenance. Since interpersonal communication is increasingly mediatized and visualized, we adopted a repertoire-oriented approach (Linke, 2011) to explore the entire communication universe of partners and friends from both individual and dyadic perspectives. Specifically, the present study adopted the concept of visual-related conflicts to understand how problematic uses of visual practices and visual communication can contribute to interpersonal conflict between partners and friends. Our results also showed that visuals can be used to resolve conflicts, although we did not expand on the positive role of visuals since this was beyond the scope of this investigation.

Our findings indicated that visual communication and visual practices, such as sharing, archiving, or looking at visuals, play an important role in engendering interpersonal conflict. Visual-related conflicts may be due to various reasons, including miscommunication during a visual exchange, failure to negotiate norms around visual online sharing, lack of or inadequate visual relational presentation on SNS profiles, unexpected discoveries while monitoring visuals shared by the other, or intrusive requests to delete visuals. Consistent with the communicative interdependence perspective (Caughlin et al., 2016), we highlighted that visual-related conflicts can arise and be managed across various communication channels. By adopting a repertoire-oriented approach, we were able to grasp and contextualize the entire process of creation and resolution of interpersonal conflict in close relationships in the maintenance phase.

Overall, we showed that the polysemic nature of images, in particular, can facilitate interpersonal conflict. Images can yield different interpretations regarding both the content of the visuals and the role attributed to the communicative act. We also saw that it is the everyday mundane images that contribute to the creation of conflict situations in the relationship. In fact, everyday photographs can be taken easily and shared instantaneously, and are increasingly exchanged and used by partners and friends for a range of communication purposes – often without careful reflection. Of course, our study focused on the maintenance phase, but we learned that partners and friends are attentive toward future changes in their relationships. For instance, they are aware that sensitive pictures, like sexting exchanges, might become problematic after a breakup. Given that close relationships are continuously evolving, other practices of visual communication may also change in their frequency and significance over time, potentially influencing the occurrence of conflicts resulting from the use of such visuals. Therefore, we suggest that future research should examine these potential changes by conducting a longitudinal study focused on visual-related conflicts in close relationships. Moreover, compared to other forms of communication, the visual modality is characterized by the greater immediacy of communication that can elicit highly intense emotions, which can exacerbate conflict situations. Finally, as in the case of conserving memories, we pointed out that images are often regarded as highly personal and that it might be difficult for visual practices to combine individual needs with relational needs. Since romantic relationships are increasingly mediatized and visualized platform affordances such as those of SNSs tend to focus on the individual rather than the relationship, such difficulty is further amplified. Overall, it must be stressed that not all the study participants reported the occurrence of visual-related conflict. Some couples with a rather ‘fragile’ relationship biography, characterized by multiple breakups and extra-dyadic affairs, did refer to more serious controversies or conflicts regarding visuals. Nevertheless, most dyads reported minor misunderstandings based on visuals and were hesitant to call them conflicts; instead, they spoke extensively about the positive aspects of visual communication, underlining its beneficial role in maintaining close bonds.

Our study comes with some limitations. First, we focused on a sample based on a specific geographical area. This could have impacted our study in various ways. For example, compared to a study that investigated the use of technology in UK and US contexts (Barassi, 2020), we observed considerably lower levels of surveillance in everyday practices. Future research could consider expanding our study by investigating visual-related conflicts in different cultural regions to grasp if and how cultural norms of visual communication could eventually play a role. Additionally, some visual-related conflicts involved specific communication channels (e.g. SNSs) that were not adopted by all the participants. We recommend that future research focus more closely on SNSs as a source of visual-related conflicts in close dyadic relationships. Moreover, our study identified specific visual-related situations in which interpersonal conflict arose. We argue that future research could adopt a mixed-methods methodological design to deepen knowledge about visual-related conflicts by comparing the frequency and perceived severity of the different visual-related situations identified herein.

Furthermore, we did not include teenagers under 18 due to the study focus on a potentially sensitive topic. However, since SNSs were involved in several visual-related conflicts and recent worldwide reports have highlighted the large number of daily underage users of such platforms, future research should also consider including teenagers in their studies. Despite these limitations, we believe that the present study sheds light on the role of visual communication and visual practices as sources of conflict in close relationships.

## Supplemental Material

sj-docx-1-vcj-10.1177_14703572231213936 – Supplemental material for Visual-related conflicts in close relationships Supplemental material, sj-docx-1-vcj-10.1177_14703572231213936 for Visual-related conflicts in close relationships by Federico Lucchesi and Katharina Lobinger in Visual Communication

## Notes

1. For further information about participants, see Lobinger et al. (2024).

2. An Italian expression, meaning ‘*confusing one thing for another*’.

3. All quotes have been translated into English from Italian, French or (Swiss) German.

4. The respondents’ age and gender are mentioned only in the first instance.

5. By ‘couple picture’, we refer to dyadic pictures that can include both partners and friends.
