# Supplementary material for: Visual-related conflicts in close relationships
Source: Vis Commun. 2024 Mar 8;24(4):808–34. doi: 10.1177/14703572231213936 (PMC12588420; doi:10.1177/14703572231213936)
Supplement: sj-docx-1-vcj-10.1177_14703572231213936 – Supplemental material for Visual-related conflicts in close relationships [file sj-docx-1-vcj-10.1177_14703572231213936.docx]

**Appendix**

| **Table 1** | | | | |
| --- | --- | --- | --- | --- |
| *Participants Overview* | | | | |
| **ID and Anonymized Names** | **Gender** | **Age** | **Type & duration of relationship** | **Living situation** |
| G01 (Hannah & Timo) | F/M | 18/18 | Couple, 2 years | Living apart |
| G02 (Cara & Mike) | F/M | 37/54 | Couple, 4 years | Living apart |
| I03 (Marika & Tommaso) | F/M | 33/33 | Married, 12 years | Living together |
| I04 (Dennis & Valentina) | F/M | 35/30 | Married, 17 years | Living together |
| F05 (Zoé & Lucas | F/M | 28/28 | Couple, 2 years | Living together |
| F06 (Manon & Hugo) | F/M | 32/31 | Couple, 3.5 years | Living together |
| I07 (Carolina & Matteo) | F/M | 19/19 | Couple, 3 years | Living apart |
| I08 (Maurizio & Vanessa) | M/F | 20/20 | Friends, 7 years | Living apart |
| I09 (Derrick & Pietro) | M/M | 25/25 | Friends, 1.5 years | Living apart |
| I10 (Diego & Marianna) | M/F | 32/31 | Married, 6,5 years | Living together |
| G11 (Raul & Tobias) | M/M | 24/22 | Couple, 1.5 years | Living together |
| G12 (Lily & Nathan) | F/M | 23/25 | Couple, 7.5 years | Living together |
| G13 (Fiona & Kim) | F/F | 26/24 | Couple, 1.5 years | Living apart |
| G14 (Anita & Gaby) | F/F | 56/55 | Friends, 36 years | Living apart |
| G15 (Mario & Vincent) | M/M | 52/47 | Friends, 22 years | Living apart |
| G16 (Nathalie & Verena) | F/F | 36/41 | Friends, 16 years | Living apart |
| G17 (Gloria & Martin) | F/M | 69/69 | Married, 14 years | Living together |
| I18 (Adelaide & Nola) | F/F | 20/20 | Friends, 4 years | Living apart |
| F19 (Liesel & Fernand) | F/M | 80/91 | Married, 50 years | Living together |
| F20 (Jade & Loic) | F/M | 51/50 | Friends, 6 years | Living apart |
| F21 (Urs & René) | M/M | 55/53 | Friends, 30 years | Living apart |
| F22 (Madeleine & Pierre) | F/M | 62/61 | Married, 32 years | Living together |
| I23 (Natalia & Patricia) | F/F | 29/40 | Married, 3 years | Living together |
| I24 (Costanza & Alberto) | F/M | 25/25 | Couple, 3,5 years | Living together |
| I25 (Dreina & Leonardo) | F/M | 22/22 | Couple, 2,5 years | Living together |
| I26 (Filiberto & Chiara) | M/F | 23/21 | Couple, 5 years | Living apart |
| I27 (Giuseppina & Silvestro) | F/M | 35/36 | Couple, 9 years | Living together |
| I28 (Lisandra & Walter) | F/M | 58/65 | Open relationship, 9 years | Living together |
| I29 (Giorgia & Yara) | F/F | 26/25 | Friends, 10 years | Living apart |
| I30 (Alessandro & Ivan) | M/M | 35/36 | Couple, 5 years | Living together |

*Note.* All participants’ names are anonymized. The pair ID gives information about the language of the interviews (G=German; I=Italian; F=French). Relationship type and duration are based on participants’ assessment.
